# Direct molecular analysis of *Malassezia* species from the clinical samples of patients with pityriasis versicolor

**DOI:** 10.18502/CMM.2023.345029.1398

**Published:** 2023-03

**Authors:** Esmaeil Eghtedarnejad, Somayeh Khajeh, Kamiar Zomorodian, Zeinab Ghasemi, Somayeh Yazdanpanah, Marjan Motamedi

**Affiliations:** 1 Department of Medical Parasitology and Mycology, School of Medicine, Shiraz University of Medical Sciences, Shiraz, Iran; 2 Basic Sciences in Infectious Diseases Research Center, Shiraz University of Medical Sciences, Shiraz, Iran; 3 Mycology Laboratory, Razi Hospital, Tehran University of Medical Sciences, Tehran, Iran

**Keywords:** *Hha*I enzyme, *Malassezia globosa*, *Malassezia restricta*, PCR− RFLP, Tinea versicolor

## Abstract

**Background and Purpose:**

Species identification of *Malassezia* using culture-dependent methods is time-consuming due to their fastidious growth requirements. This study aimed to evaluate a rapid and accurate molecular method in order to diagnose the pityriasis versicolor (PV) and identify *Malassezia* species from direct clinical samples.

**Materials and Methods:**

Skin scraping or tape samples from patients with PV and healthy volunteers as the control group were collected. Diagnosis of PV was confirmed by direct microscopic examination. The DNA extraction was performed according to the steel-bullet beating method. Polymerase chain reaction-restriction fragment length polymorphism assay using *Hha*I restriction enzyme was applied for the identification and differentiation of *Malassezia* species.

**Results:**

The PCR method was able to detect *Malassezia* in 92.1% of specimens which were also confirmed with microscopic examination. Statistically, a significant association was observed between the results of the two assays (*P* < 0.001). Moderate agreement was identified between the two methods to diagnose the PV in both populations (Kappa: 0.55). Considering microscopic examination as the gold standard method for confirmation of PV, the sensitivity, specificity, positive predictive value, and negative predictive value values of the PCR assay for recognition of PV were 85%, 75%, 92%, and 60%, respectively. *M. globosa* and *M. restricta* were the most prevalent species isolated from patients.

**Conclusion:**

In this study, the two-step molecular method based on the amplification of the D1/D2 domain and digestion of the PCR product by one restriction enzyme was able to diagnose and identify *Malassezia* directly from clinical samples. Consequently, it can be said that the molecular-based method provides more facilities to identify fastidious species, such as *M. restricta*.

## Introduction

*Malassezia* species are lipid-dependent basidiomycetous yeasts with numerous functionally distinct strains that have recently been classified as Malasseziomycetes class. Among the different clinical manifestations of *Malassezia* species, Pityriasis versicolor (PV) is a well-known superficial infection of the skin due to overgrowth or morphogenesis of yeasts to filamentous forms [ 1 ].

During the past years, molecular-based assays [ 2 - 7 ] for *Malassezia* detection/identification have revealed the ubiquitous presence of *Malassezia* on the skin in addition to pathogenic roles in different skin disorders [ 8 ]. While these approaches have met with various degrees of success, most of them require cultivation to enhance sensitivity, which increases both the potential for cultural bias and the turnaround time for analysis.

In this regard, two culture-independent molecular methods for the detection and identification of *Malassezia* from clinical samples have been tested in recent years. However, these methods require either separate amplification with specific primer sets for each species [ 4 , 5 , 7 ] or sequencing which can be costly and time-consuming.

Therefore, the present study aimed to evaluate the rapid and accurate molecular method for the species identification of *Malassezia* directly from clinical samples by amplification of the D1/D2 domain using Polymerase chain reaction-restriction fragment length polymorphism (PCR−RFLP).

## Materials and Methods

In this cross-sectional study, samples from the skin of 125 patients with PV who referred to the Medical Mycology Laboratory of Razi Hospital in Tehran were collected. Moreover, 17 healthy volunteer individuals were included as the negative control group. The diagnosis of PV was confirmed by microscopic observation of scales using potassium hydroxide (10% KOH) examination, which demonstrated the typical ovoid clusters of yeast cells and short filamentous. Following the confirmed diagnosis of PV, a part of the sample was stored at 25 °C for molecular analysis by PCR assay.

To identify the *Malassezia* species by molecular method, fungal genomic DNA was extracted manually from each clinical sample using steel bullet disruption [ 9 ]. The D1/D2 domains of the 26S rRNA gene which produces approximately 580 bp fragments were amplified [ 6 ]. The PCR reactions were performed in a 25 μL final volume containing 2×Taq Master Mix (final conc. 1×) (Ampliqon RED, Denmark), 0.5 μM primer, and 4 μL (20 ng) of template DNA. Temperature conditions for PCR were 5 min at 95 °C, 37 cycles of 45 s at 95 °C, 45 s at 58 °C and 1 min at 72 °C, followed by 7 min at 72 °C. The PCR products were analyzed by electrophoresis on 1% (w/v) Tris–acetate–EDTA agarose gels containing 2.5-3% ethidium bromide, and visualized under a UV detector (UVITEC, UK).

The 142 PCR products of the D1/D2 domains were subjected to RFLP analysis with the *Hha*I enzyme (New England Biolabs, Beverly, MA, USA). Restriction reactions were carried out in 10 μL volumes containing 5 μL (200 ng) of PCR product, 2 μL of the restriction enzyme, 1μL of appropriate reaction buffer (final conc. 1×), and 2 μL of distilled water at 37 °C for 24 h. Restriction patterns were compared with those of reference *Malassezia* strains and species-specific patterns established in the original publications [ 6 ]. The DNA sequencing of D1/D2 domains was performed for 2 clinical samples that were confidently identified by PCR−RFLP, and for 8 clinical samples that could not be identified by PCR−RFLP. 

The Chi-squared test was used to analyze the statistical difference between the ability of methods to detect *Malassezia* species in patients and healthy subjects. The sensitivity, specificity, positive predictive value (PPV), and negative predictive value (NPV) of the PCR assay were calculated.

## Results

**Figure 1:**
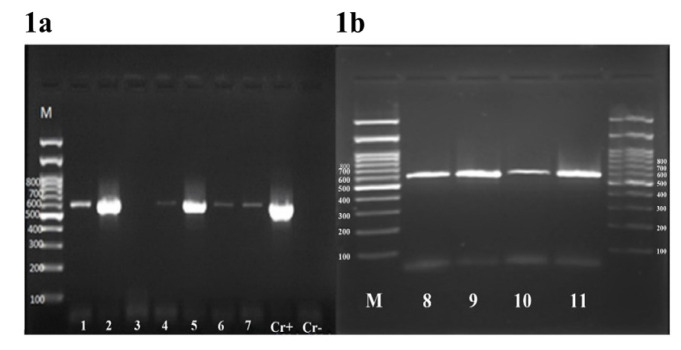
Agarose gel electrophoresis of D1/D2 region of PCR products. Before digestion with *HhaI*: lanes 1-7 are example samples. Lanes cr+ and cr- are positive and negative controls, respectively, and lane M is a 100-bp molecular size marker. **Figure 1B:** After digestion with *HhaI*: lanes 1 and 2 are *M. globosa*, lanes 3 and 4 are *M. restricta*, and lane M is a 100-bp molecular size marker.

This study was performed on 142 participants from whom 104 (73.2 %) skin scales and 38 (26.8%) scotch tapes were collected. The PCR amplification of the partial D1/D2 region produced a single amplicon of the expected size (Figure 1A) for 102 samples. In comparison to a molecular analysis by PCR, more samples were positive by direct microscopic examination (102 vs. 110). The PCR method could detect *Malassezia* in 94 (92.1%) specimens which were also positive in microscopic examination. 

Moreover, the PCR analysis detected *Malassezia* in eight samples that showed negative results using the direct microscopy method. Statistically, a significant association was observed between the results of the two assays (*P*<0.001). Considering microscopic examination as the gold standard method for confirmation of PV, the diagnostic performance of the PCR assay for recognition of PV was calculated. The sensitivity, specificity, PPV, and NPV values of the PCR were 85%, 75%, 92%, and 60%, respectively.

Upon digestion of the amplified products with the *Hha*I restriction enzyme, two different restriction patterns could be distinguished. Both patterns matched exactly the *Hha*I restriction patterns predicted for *Malassezia globosa* (129 and 455 bp), and *Malassezia restricta* (580 bp). In line with these patterns, *M. globosa* and *M. restricta* were detected in 80 (85.1%) and 14 (14.9%) samples, respectively (Figure 1B).

Based on the sequencing data of the D1/D2 region for eight clinical samples that could not be distinguished by PCR−RFLP, *M. globosa* and *M. restricta* were identified with 100% identity in seven and one samples, respectively. Representative sequences of the DNA-sequenced *Malassezia* are deposited in the GenBank database with accession numbers of OR234714-OR234723. 

The results of species distribution in the study population showed that *M. globosa* was found in 67.7% (n=84) of the patients with PV, and 11.7% (n=2) of the healthy population. It should be noted that *M. restricta* was just identified in patients with PV (15; 12.1%).

## Discussion

According to the results, molecular identification involving PCR−RFLP analysis had a remarkable ability for the direct identification of *Malassezia* in clinical samples. As can be seen in previous studies [ 10 , 11 ], the diagnosis of fungal infections using PCR-based methods, if performed directly on clinical samples, has a significant improvement, compared to the results obtained from the conventional methods. 

The advantages of these methods include 1) the possibility of simultaneous detection and identification of the causative agent, 2) no need for culture in cases where the sample cannot be cultured for any reason, and 3) reducing the time required to obtain the result to one day. Prompt detection and identification of the causative agents of infection lead to the selection of the appropriate treatment by saving time, as well as the prevention of the induction of drug resistance. Therefore, in this study, it was attempted not only to diagnose PV infection directly from the clinical sample by PCR−RFLP method but also to identify the common *Malassezia* species that are the causative agents of this infection.

The PCR assay used in this study has high diagnostic characteristics, especially in terms of sensitivity and PPV that could be arising from the selection of suitable primers for PCR analysis. Regarding species identification of *Malassezia*, the general or species-specific primers are designed from different regions of the rRNA complexes so far [ 12 , 13 ] or the *elongation factor 1 alpha* genes [ 14 ]. Consistent with previously reported results [ 15 ], the primers used in the current study amplified the D1/D2 region that was efficient for species identification of *Malassezia* yeasts.

Until now, many molecular methods have been introduced for the detection and identification of *Malassezia* species from clinical isolates. The most common methods include nested PCR [ 16 ], PCR−RFLP [ 17 ], and real-time PCR [ 3 ] with different levels of accuracy. Among the above-mentioned methods, the PCR−RFLP method is a sensitive, accurate, and fast (when using fast digest enzymes) method without requiring advanced tools that can detect microorganisms at the species and intra-species levels [ 18 , 19 ]. 

Among the other advantages of PCR−RFLP assay, it can be pointed out that there is no need for complete gene sequence information, no influence from the environmental agents, and high reproducibility [ 20 ]. However, it should be noted that this method also has disadvantages, which include the high cost of some endonuclease enzymes and the reduction of sequencing accuracy if there is more than one mutation in the gene sequence detected by the enzyme. 

It should be noted that some studies have used the D1/D2 domain for PCR−RFLP assays and identified *Malassezia* species by exposing the PCR product to two to three enzymes. It seems that the reason for the variety of species identified in the prior studies, compared to our study, is the use of more than one enzyme in the PCR−RFLP reaction.

In some studies, in order to identify *Malassezia* species, the isoschizomers of *Hha*I enzymes have been used to create breaks in the D1/D2 domain [ 6 , 21 , 22 ]. The *Hha*I has isoschizomers, such as *Cfo*I, *FnuD*III, *HinGU*I, *HinPl*I, *HinSl*I, *HinS2*I, *Mnn*IV, and *SciN*I [ 23 ].

Recent studies based on culture methods in different geographical regions of Iran have shown that *M. globosa* is the most prevalent species recovered from PV patients (frequency range: 36-43%) [ 24 - 27 ]. In view of these obtained results, it should be noted that *M. restricta* species could not easily recover by conventional culture methods. However, the independent culture methods are capable of identification of *M. restricta*. Consequently, it is worth noting that molecular methods are more efficient to determine the species distribution of fungal agents in epidemiological studies [ 5 , 28 ].

## Conclusion

In this study, the two-step molecular method based on the amplification of the D1/D2 domain and digestion of the PCR product by one restriction enzyme was able to identify *Malassezia* directly from clinical samples. Moreover, the suggested molecular-based method provides more facilities to identify fastidious species, such as *M. restricta*.

## Acknowledgments

This study was derived from a thesis submitted for partial fulfillment of the Master’s degree by Somayeh Khaje and supported by the Research and Technology Deputy of Shiraz University of Medical Sciences, Shiraz, Iran.

## Authors’ contribution

M. M. contributed to the design of the study and writing of the research paper. Z. G. performed the clinical sample collection. S. K. performed the experiments. E. E. and S. Y. contributed to the acquisition, analysis, and interpretation of data. K. Z. advised the whole process of the study. All authors provided critical revisions for important intellectual content and also read and approved the final manuscript.

## Conflicts of interest

The authors declare no conflict of interest.

## Financial disclosure

This study was financially supported by Shiraz University of Medical Sciences, Shiraz, Iran (Grant No. 25242).

## References

[1] Karray M, McKinney WP ( 2021). Tinea versicolor.

[2] Gupta AK, Ahmad I, Borst I, Summerbell RC ( 2000). Detection of xanthomegnin in epidermal materials infected with Trichophyton rubrum. J Invest Dermatol.

[3] Ilahi A, Hadrich I, Neji S, Trabelsi H, Makni F, Ayadi A ( 2017). Real-time PCR identification of six Malassezia species. Curr Microbiol.

[4] Sugita T, Suto H, Unno T, Tsuboi R, Ogawa H, Shinoda T, et al ( 2001). Molecular analysis of Malassezia microflora on the skin of atopic dermatitis patients and healthy subjects. J Clin Microbiol.

[5] Gholami M, Mokhtari F, Mohammadi R ( 2020). Identification of Malassezia species using direct PCR-sequencing on clinical samples from patients with pityriasis versicolor and seborrheic dermatitis. Curr Med Mycol.

[6] Mirhendi H, Makimura K, Zomorodian K, Yamada T, Sugita T, Yamaguchi H ( 2005). A simple PCR-RFLP method for identification and differentiation of 11 Malassezia species. J Microbiol Methods.

[7] Paulino LC, Tseng C-H, Blaser MJ ( 2008). Analysis of Malassezia microbiota in healthy superficial human skin and in psoriatic lesions by multiplex real-time PCR. FEMS Yeast Res.

[8] LeibundGut-Landmann S, Dawson Jr TL ( 2021). Malassezia: a skin commensal yeast impacting both health and disease. Front Cell Infect Microbiol.

[9] Motamedi M, Amini A, Yazdanpanah S, Mahmoodi M, Khodadadi H, Zalpoor H ( 2022). Evaluation of different DNA extraction methods based on steel‐bullet beating for molecular diagnosis of onychomycosis. J Clin Lab Anal.

[10] Perlin DS, Wiederhold NP ( 2017). Culture-independent molecular methods for detection of antifungal resistance mechanisms and fungal identification. J Infect Dis.

[11] Zhao Y, Garnaud C, Brenier-Pinchart MP, Thiébaut-Bertrand A, Saint-Raymond C, Camara B, et al ( 2016). Direct molecular diagnosis of aspergillosis and CYP51A profiling from respiratory samples of French patients. Front Microbiol.

[12] Sugita T, Takeo K, Hama K, Virtudazo E, Takashima M, Nishikawa A, et al ( 2005). DNA sequence diversity of intergenic spacer 1 region in the non-lipid-dependent species Malassezia pachydermatis isolated from animals. Medical mycology.

[13] Pérez-Pérez L, Pereiro M, Toribio J ( 2010). Classification of Yeasts of the Genus Malassezia by Sequencing of the ITS and D1/D2 Regions of DNA.

[14] Barik BP, Tayung K ( 2012). Molecular differentiation of Fusarium spp. with varied lifestyles based on TEF 1 alpha gene sequence analysis. Interdiscip Sci.

[15] Nabili M, Moazeni M, Taghizadeh Armaki M, Asgari MR, Nosrati A, Shokohi T ( 2013). Diagnostic tools in fungal infections since classical to molecular era. J Mazandaran Univ Med Sci.

[16] Jusuf NK, Nasution TA, Ullyana S ( 2018). Diagnostic value of nested-PCR for identification of Malassezia species in dandruff.

[17] Bafghi M, Mozafari NA, Fata A, Naseri A, Zarrinfar H ( 2017). Identification of Malassezia species using PCR-RFLP molecular method in the patients with pityriasis versicolor. SJKU.

[18] Pramunadipta S, Widiastuti A, Wibowo A, Suga H, Priyatmojo A ( 2022). Development of PCR-RFLP technique for identify several members of Fusarium incarnatum-equiseti species complex and Fusarium fujikuroi species complex. Plant Pathol J.

[19] Wu Z, Nagano I, Boonmars T, Nakada T, Takahashi Y ( 2003). Intraspecies polymorphism of Cryptosporidium parvum revealed by PCR-restriction fragment length polymorphism (RFLP) and RFLP-single-strand conformational polymorphism analyses. Appl Environ Microbiol.

[20] Young N, Tanksley S ( 1989). Restriction fragment length polymorphism maps and the concept of graphical genotypes. Theor Appl Genet.

[21] Gaitanis G, Velegraki A, Frangoulis E, Mitroussia A, Tsigonia A, Tzimogianni A, et al ( 2002). Identification of Malassezia species from patient skin scales by PCR-RFLP. Clin Microbiol Infect.

[22] Oh BH, Song YC, Lee YW, Choe YB, Ahn KJ ( 2009). Comparison of nested PCR and RFLP for identification and classification of Malassezia yeasts from healthy human skin. Ann Dermatol.

[23] Roberts RJ ( 1987). Restriction enzymes and their isoschizomers. Nucleic Acids Res.

[24] Moniri R, Nazeri M, Amiri S, Asghari B ( 2009). Isolation and identification of Malassezia spp. In pytiriasis versicolor in Kashan, Iran. Pak J Med Sci.

[25] Jafari AA, Zarrinfar H, Mirzaei F, Katiraee F ( 2013). Distribution of Malassezia species in patients with pityriasis versicolor compared with healthy individuals in Yazd, Iran. Jundishapur J Microbiol.

[26] Zeinali E, Sadeghi G, Yazdinia F, Shams-Ghahfarokhi M, Razzaghi-Abyaneh M ( 2014). Clinical and epidemiological features of the genus Malassezia in Iran. I Iran J Microbiol.

[27] Shams M, Rasaee MJ, Moosavi M, Razzaghi M ( 2001). Identification of Malassezia species in patients with pityriasis versicolor submitted to the Razi Hospital in Tehran. IBJ.

[28] Mahmoudabadi AZ, Zarrin M, Azish M ( 2014). Detection of Malassezia species isolated from patients with pityriasis versicolor and seborrheic dermatitis using Nested-PCR. Jundishapur J Microbiol.

